# Production of cross neutralizing single chain fragment variables (scFv) from HIV-1 infected Indian children

**DOI:** 10.1186/1471-2334-14-S3-E25

**Published:** 2014-05-27

**Authors:** Sanjeev Kumar, Rajesh Kumar, Muzamil Makhdoomi, Lubina Khan, SS Prakash, Ramachandran Thiruvengadam, Mohit Singla, Rakesh Lodha, SK Kabra, Subrata Sinha, Kalpana Luthra

**Affiliations:** 1 Department of Biochemistry, All India Institute of Medical Sciences (AIIMS), New Delhi, India; 2 Department of Pediatrics, All India Institute of Medical Sciences (AIIMS), New Delhi, India; 3 National Brain Research Centre (NBRC), Gurgaon, Haryana, India

## Background

Monoclonal antibody based vaccines are effective and highly specific in disease targeting. Presently, most of the existing broadly neutralizing antibodies are generated against non subtype c viruses. HIV-1 subtype c accounts for more than 90% of infection in India. The disease progression in children is faster than adults.

## Methods

Nine ART drug naïve HIV-1 subtype c infected children were recruited. PBMCs were isolated from all the subjects and pooled. RNA was isolated and cDNA was synthesized followed by amplification of VH and VL chain genes and scFv construction. A human recombinant scFv phage display library of 108 clones was constructed. Diversity of the phage library was checked by DNA sequencing and biopanned with RSC3 core antigen. 60 random clones were screened by phage ELISA. Expression of the scFvs was assessed by SDS-PAGE and Western blotting.

## Results

The diversity of the phage library was more than 90%. Eight scFv monoclonals showed positive binding in phage ELISA and two best binders were further characterized. Both scFvs didn’t show any reactivity with unrelated antigens. DNA fingerprinting analysis showed both scFvs were distinct. A 32kDa band was obtained in SDS-PAGE and Western blot. Both scFvs showed cross neutralizing activity against a standard panel of pseudoviruses.

## Conclusion

Here, we have for the first time generated a human recombinant scFv phage display library containing neutralizing clones from HIV-1 infected children. Further characterization of these scFvs generated against CD4 binding site for their epitope mapping would be helpful in the development of an effective HIV-1 vaccine.

